# Sex-Based Difference in Aortic Dissection Outcomes: A Multicenter Study

**DOI:** 10.3390/jcdd10040147

**Published:** 2023-03-30

**Authors:** Francesco Nappi, Sandra Petiot, Antonio Salsano, Sanjeet Singh Avtaar Singh, Joelle Berger, Marisa Kostantinou, Severine Bonnet, Ivancarmine Gambardella, Fausto Biancari, Almothana Almazil, Francesco Santini, Rim Chaara, Antonio Fiore

**Affiliations:** 1 Department of Cardiac Surgery, Centre Cardiologique du Nord, 93200 Saint Denis, France; 2 Department of Anesthesia, Centre Cardiologique du Nord, 93200 Saint Denis, France; 3 Division of Cardiac Surgery, Ospedale Policlinico San Martino, DISC Department, University of Genoa, 16126 Genoa, Italy; 4 Department of Cardiothoracic Surgery, Royal Infirmary of Edinburgh, Edinburgh EH16 4SA, UK; 5 Department of Cardiothoracic Surgery, Presbyterian Medical Center, 505 E 70th St., New York, NY 10065, USA; 6 Heart and Lung Center, Helsinki University Hospital, University of Helsinki, 00231 Helsinki, Finland; 7 Department of Cardiac Surgery, Hôpitaux Universitaires Henri Mondor, Assistance Publique-Hôpitaux de Paris, 94000 Creteil, France

**Keywords:** aortic dissection, ascending aorta replacement, conservative surgery, total arch replacement procedure, mesenteric ischemia

## Abstract

Background: Type A Acute Aortic Dissection (TAAAD) repair is a surgical emergency associated with high morbidity and mortality. Registry data have noted several sex-specific differences in presentation with TAAAD which may account for the differences in men and women undergoing surgery for this condition. Methods: A retrospective review of data from three departments of cardiac surgery (Centre Cardiologique du Nord, Henri-Mondor University Hospital, San Martino University Hospital, Genoa) between January 2005 and 31 December 2021 was conducted. Confounders were adjusted using doubly robust regression models, a combination of regression models with inverse probability treatment weighting by propensity score. Results: 633 patients were included in the study, of which 192 (30.3%) were women. Women were significantly older with reduced haemoglobin levels and pre-operative estimated glomerular filtration rate compared to men. Male patients were more likely to undergo aortic root replacement and partial or total arch repair. Operative mortality (OR 0.745, 95% CI: 0.491–1.130) and early postoperative neurological complication results were comparable between the groups. The adjusted survival curves using IPTW by propensity score confirmed the absence of a significant impact of gender on long-term survival (HR 0.883, 95% CI 0.561–1.198). In a subgroup analysis of women, preoperative levels of arterial lactate (OR 1.468, 95% CI: 1.133–1.901) and mesenteric ischemia after surgery (OR 32.742, 95% CI: 3.361–319.017) were significantly associated with increased operative mortality. Conclusions: The advancing age of female patients alongside raised preoperative level of arterial lactate may account for the increasing preponderance among surgeons to perform more conservative surgery compared to their younger male counterparts although postoperative survival was similar between the groups.

## 1. Introduction

Recent improvements in perioperative care and surgical techniques have significantly improved outcomes of Type A Acute Aortic Dissection (TAAAD) repair, but it remains a surgical emergency associated with high morbidity and mortality [1,2,3,4].

Several sex-specific differences have been described in TAAAD. Women have a lower incidence, present at a later age, and present with atypical findings, despite the frequency in men being two-fold higher. More than 50% of women experienced Type A Acute Aortic Dissection after the age of 70 years and diagnosis is often delayed due to the absence of specific symptoms and signs [5,6,7,8,9]. These findings may contribute to poorer outcomes and higher mortality of TAAAD repair in women, as shown in several studies [9,10,11]. In contrast, other studies showed no sex differences in inpatient or 30-day mortality or in outcomes, probably due to meticulous Type A Acute Aortic Dissection management in women [5,6,7,12,13,14]. Other differences are in the extension of the dissected aorta that is often limited with less visceral and renal malperfusion in women, alongside reduced frequency of aortic root involvement resulting in a shorter operation time [7,12].

In this study, we investigate the outcomes and mortality after surgery for acute Type A Acute Aortic Dissection in women and we analyze if aortic arch repair and hypothermic cardiac arrest times are predictive of mortality.

## 2. Methods

### 2.1. Study Design and Oversight, Patient Population, Definitions, and Outcomes

Data were gathered from three departments of cardiac surgery (Centre Cardiologique du Nord, Henri-Mondor University Hospital, San Martino University Hospital, Genoa, Italy) between January 2005 and 31 December 2021 and examined retrospectively. The databases were audited by clinical information analysts within each unit and were confirmed periodically with internal and external controls. Preoperative and postoperative variables were incorporated retrospectively including hospital stay and follow-up data which were updated each year. Follow-up data for survival were updated by correspondence with referring physicians, or direct phone contact with patients and family. This study was approved by the institutional review board (Approval Number assigned by the IRB: IRB-MTP_2022_07_202201173) and written informed consent was provided by all patients.

### 2.2. Patients and Outcomes

A total of 633 patients were identified from the database with baseline data, demographics, and follow-up data inspected. Inclusion criteria for this study were Type A Acute Aortic Dissection or intramural hematoma involving the ascending aorta and patients aged >18 years, symptoms within 7 days from surgery, primary surgical repair of type A acute aortic dissections, any other major cardiac surgical procedure concomitant with surgery for TAAAD, and retrograde TAAAD with primary tear detected in the descending aorta. Exclusion criteria consisted of patients aged <18 years, onset of symptoms >7 days from surgery, a previous procedure for Type A Acute Aortic Dissection, concomitant endocarditis, and TAAAD following blunt or penetrating chest trauma.

Outcomes included both in-hospital complications and late survival. In-hospital outcomes were: stroke, paraplegia, tetraplegia or tetraparesis, laryngeal nerve palsy, mesenteric ischemia, sepsis, acute renal failure requiring dialysis, atrial fibrillation, reoperation for intrathoracic bleeding, deep sternal wound infection or mediastinitis, intra-aortic balloon pump (IABP), venoarterial extracorporeal membrane oxygenation (VA ECMO), ICU stay, and operative mortality (OM). OM is defined as 30-day and in-hospital mortality.

### 2.3. Surgical Technique

All procedures were performed via median sternotomy. The referring surgeon at each site dictated the surgical procedure regarding the preferred site for cannulation, which in the majority of patients was performed in the innominate artery, right femoral artery, axillary artery, or central aortic lumen. Cardiopulmonary bypass management and the degree of systemic cooling once the patients were positioned for surgery were also surgeon-specific. Diastolic arrest was achieved by direct delivery of antegrade potassium-rich cardioplegia solution into the coronary ostium or the coronary sinus cannula insertion in patients with aortic regurgitation or when radical procedures and/or extensive aortic arch repairs were planned. The resection of the ascending aorta was extended up to the sinotubular junction. The thrombus identified in the false lumen of the aortic root was removed so that the aortic lesion could be visualized. Then, after inspecting the anatomy of the root and evaluating the state of the aortic valve leaflets, the intima was re-approached to the adventitia. Resuspension of the commissures was routinely performed using 4–0 or 5–0 sutures reinforced with a Teflon pledget over each commissure. The handling of commissures was performed in patients receiving valve-sparing root procedures and in recipients of ascending aortic root-sparing replacement where a subsequent commissural collapse was caused by intimal separation extending up to the sinuses. In general, the use of biologic glue neo-media during reconstruction was routine while the use of felt was dictated by the individual surgeons’ habits. The proximal anastomosis was made using a 4–0 or 5–0 polypropylene suture, and this suture line also secured the intima to the adventitia. To achieve an uninterrupted external ring of felt reinforcement, the use of felt neo-media or an overlay of horizontal felt-mattress sutures was positioned circumferentially and dictated by the surgeon’s predilection. The replacement of the aortic root using a biologic or mechanical composite valve graft or valve-sparing root reimplantation procedure was recommended in patients who disclosed dilatation of the sinuses of Valsalva >4.5 cm in diameter on computed tomography imaging, those with connective tissue disorders, or those in whom intimal tears extended into the Valsalva sinuses. In contrast, in patients with normal-sized aortic roots associated with poor-quality valve leaflets, concomitant aortic valve replacement with the use of conventional xenograft or mechanical prosthesis was favored.

Total arch replacement procedures (TARP) were achieved using deep hypothermic circulatory arrest and with either antegrade or retrograde cerebral perfusion, preserving systemic cooling between 19 °C to 25 °C, contingent on the surgeon’s habits. Symmetric brain cooling and warming were monitored through the use of near-infrared spectroscopy which confirmed symmetric brain cooling and heating. The technique of cerebral protection, type of cannulation, and technique of perfusion were chosen according to the discrimination of the surgeon. In the majority of patients, antegrade cerebral protection was delivered using endoluminal technique or direct cannulation of the right axillary artery or the innominate trunk or common carotid. The flow rate injected was 800–1000 mL/min at 28 °C or 36 °C while maintaining systemic pressure between 40 and 60 mmHg. In the remaining 19.5% of cases undergoing arch repair, the procedure was performed by delivering retrograde cerebral perfusion in the condition of deep hypothermic circulatory arrest. Cerebroplegia was administered by a cannula inserted into the superior vena cava and delivered at 200–350 mL/min at 18 °C with central venous pressure maintained between 25 and 35 mmHg.

One- and four-branch prostheses were preferred in patients receiving a TARP procedure that involved the resection of all the aortic tissue up to the left common carotid artery (total hemiarch) or reimplantation of the innominate trunk only (partial hemiarch). TARP that required large vessel reimplantation was instead addressed in patients with a large arch aneurysm or extensive intimal lesion within the arch. The surgical option to perform arch debranching and selective vessel implantation was preferred in patients with connective tissue disorders or significant dislocation of the great vessels. The patients who needed a frozen elephant trunk procedure underwent either insular replantation or selective debranching/implantation of vessels. Antegrade cardiopulmonary bypass was reinstituted using a lateral reperfusion branch of the graft. Systemic warming was performed while preserving a temperature gradient of 10 °C between the blood and the core temperature during hemostasis. The remaining anastomoses were completed and reinforced according to the previously described technique and the cardiopulmonary bypass was stopped when the core body temperature reached 36 °C.

### 2.4. Statistical Analysis

Categorical data were presented as frequencies and percentages and compared using the Chi-square test or Fisher’s exact test where appropriate. Continuous variables were expressed as median and interquartile range [IQR] and compared using a two-tailed Mann–Whitney test for non-parametric distributions. Trends in surgical procedures over time were tested by applying the sieve bootstrapped t-test. To adjust for confounding, a doubly robust method (a combination regression model with inverse probability treatment weighting (IPTW) by propensity score) was used to estimate the causal effect of the exposure on the outcomes [15]. For this purpose, a covariate balancing propensity score (CBPS) was developed to minimize the differences between sexes [16]. The full list of these covariates is given in the Supplementary Table S1. Using the estimated propensity scores as weights, an inverse probability weighting (IPW) model was used to generate a weighted cohort [17]. C-statistics were calculated to ascertain the validity of the propensity score. The long-term mortality in female and male patients was assessed using the Kaplan–Meier survival curves and IPTW by propensity score to build adjusted survival curves [18].

Subgroup analysis for predictors of operative mortality in women was conducted. Variables significantly associated (*p* < 0.05) with death at univariate analysis were included in a parsimonious multivariable stepwise logistic regression model with selection based on the Akaike information criterion (AIC). Results were reported as odds ratio (OR), 95% confidence limits (95% CI), and *p*-value. The final model was internally validated using 1000 bootstrapping iterations. The receiver operating characteristic (ROC) curve was used to estimate the discrimination power. Calibration was assessed through the Hosmer–Lemeshow test.

Statistical analyses were performed using R software (version 4.2.1; R Foundation for Statistical Computing, Vienna, Austria).

## 3. Results

### 3.1. Baseline

Overall, 633 consecutive patients underwent surgery for type A acute aortic dissection from January 2005 to December 2022. Among these, 192 (30.3%) were females and 441 (69.7%) were males. Table 1 summarizes the baseline characteristics between sexes after combining operative data.

**Figure 1:**
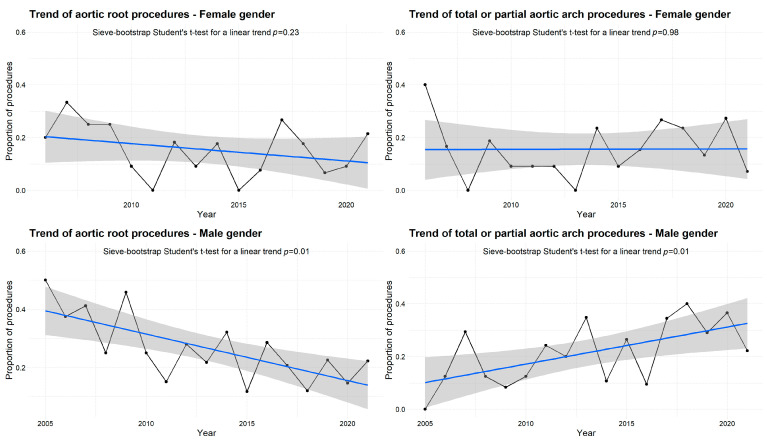
Trends in surgical procedures in male and female patients.

Women were significantly older and had reduced weight, height, and haemoglobin levels compared to men as expected. The estimated glomerular filtration rate (eGFR) was significantly lower in females. Bicuspid aortic valves were more frequently found in male patients. Compared to women, male patients were more likely to undergo aortic root replacement and partial or total arch repair. The proportion of the procedures on the aortic root decreased gradually over the study period (*p* for trend = 0.01, Figure 1), while the proportion of arch surgery increased gradually across the years (*p* for trend = 0.01, Figure 1) in men. On the other hand, among female patients, the proportions of surgical procedures did not change significantly over time (Figure 1).

### 3.2. Postoperative Results

Table 2 demonstrates the sex difference of the early outcomes before (crude rates) and after doubly robust adjustment.

As shown in the Supplementary Table S1 and Supplementary Figures S1 and S2, all the covariates of the weighted cohort were well balanced between groups apart from the variable “bicuspid valve” which was taken into account in the final statistical models. The C-statistics of the propensity score was 0.709 (Supplementary Figure S3). The regression models demonstrated that women had a significantly lower risk of postoperative acute renal failure requiring dialysis (OR 0.504; 95% CI, 0.271–0.939; *p* = 0.031).

Early postoperative neurological complications (Stroke 13% vs. 13%, Paraplegia 4.7% vs. 3.2% for women and men, respectively) and operative mortality (overall 25% of which 27% for women vs. 24% for men, doubly robust OR 0.745, 95% CI: 0.491–1.130, *p* = 0.166) were comparable between groups. Early mortality rates varied during the study period without significant change over the years (Sieve-bootstrap Student’s *t*-test for a linear trend, *t* value = −0.86368, *p*-value = 0.496, Supplementary Figure S4).

**Figure 2:**
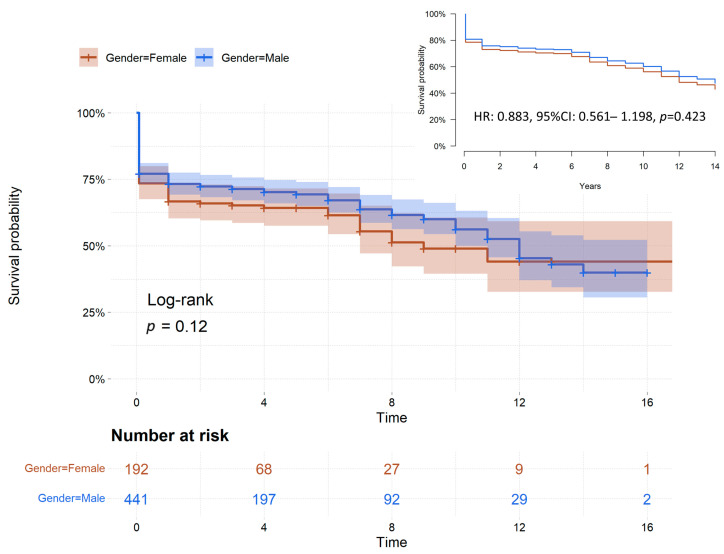
Kaplan–Meier survival curves stratified by gender and adjusted survival curves. CI, confidence interval; HR, hazard ratio.

The mean follow-up was 3.8 years ± 4.02 months (median 2 years; interquartile range: 1–6 years) and overall survival rates at 1, 5, and 10 years were 71.2% ± 2.5%, 67.7 ± 2.9%, 56.8 ± 4.6%. Actuarial survival after surgery at 1, 5, and 10 years were 66.7 ± 5.1%, 64.2 ± 5.6%, 48.9 ± 10.9% vs. 73.2 ± 2.9%, 67.1 ± 3.6%, 56.2 ± 6.0% for female and male patients, respectively. The two survival curves were not significantly different when compared using a log-rank test (*p* = 0.12, Figure 2). The adjusted survival curves using IPTW by propensity score confirmed the absence of a significant impact of gender on long-term survival (HR 0.883, 95% CI 0.561–1.198, *p* = 0.423, Figure 2).

### 3.3. Subgroup Analysis: Predictors of Operative Mortality in Female Patients

Operative mortality was slightly higher in women (26.6%, Table 1 and Table 3) compared to men but was not statistically different when the entry tear of the dissection was identified in the ascending aorta (25.0% vs. 27.6%, *p* = 0.693), aortic root (26.5% vs. 26.7%, *p* = 0.986), or aortic arch (26.2% vs. 30%, *p* = 0.712), and in the case of surgery involving the aortic root (27.6% vs. 20.7%, *p* = 0.440) or partial/total repair of the aortic arch (27.2% vs. 23.3%, *p* = 0.665).

The results of univariable and multivariable analyses of factors associated with in-hospital mortality are reported in Table 3.

At multivariable analysis, preoperative level of arterial lactate (OR 1.468, 95% CI: 1.133–1.901, *p* = 0.004) and mesenteric ischemia after surgery (OR 32.742, 95% CI:3.361–319.017, *p* = 0.003) were significantly associated with increased operative mortality. On the other hand, eGFR (OR 0.952, 95% CI: 0.929–0.976, *p* < 0.001) was significantly associated with reduced operative mortality.

The C-statistics of the multivariable model was 0.849 and Hosmer and Lemeshow Goodness-of-Fit test *p* value was 0.80.

Practical information for clinicians: using the beta-coefficient and constant reported in the Table 3, the risk model should become according to the following formula: exp [0.884 + (Arterial lactate × 0.384) + 3.489 in case of mesenteric ischemia+ (eGFR × −0.049)].

## 4. Discussion

The results of this 16-year study (2005–2021) advanced our understanding of the relative benefits of aortic surgery for the management of acute aortic dissection type A in women. Patient characteristics and surgical strategies including annual case volumes were collected. We observed that a greater number of overall patients were managed with ‘conservative’ procedures undergoing replacement of the ascending aorta procedure, using the interposition of dacron prosthesis combined with/without replacement of the aortic root and the arch replacement. Comparatively, in men, the proportion of patients who received surgery with the involvement of aortic root decreased gradually over the study period while the proportion of subjects who underwent arch surgery increased gradually across the years (Figure 1). Conversely, in women, there was not a substantial variation (Figure 1) and fewer women underwent associated ascending aorta and arch repair with/without root replacement. The observed overall rates of operative death were 25%. Although in our series the women were significantly older, the operative mortality rate was comparable between groups (women 27% vs. men 24% *p* = 0.166) without significant changes across years. We identified three independent risk factors associated with mortality: arterial lactate, mesenteric ischemia after surgery, and estimated glomerular filtration rate. At the mean follow-up of 3.8 years, no significant differences were observed between groups in the rank-based assessment of death and survival was similar at the 10-year follow-up (48.9% in the women and 56.2% in the men). In our series, although survival curves adjusted with the use of inverse probability treatment weighting by propensity score confirmed the equivalent impact of gender on long-term survival, it was not powered to draw firm conclusions about the relative effects of aortic surgery on survival among men and women affected by TAAAD.

Our results contradict much of the published literature on this topic, which reports several disadvantages in women compared to Type A Acute Aortic Dissection repair in men, including higher operative mortality, neurological outcomes, and lower long-term survival rates [9,10,11,13]. The last report of the IRAD registry (International Registry of Acute Aortic Dissection) evaluated 2823 patients of which 34.3% were women. Poorer early surgical outcomes were noted in women compared to men (*p* = 0.039) with higher in-hospital mortality (16.7% vs. 13.8%) despite the similar delay, surgical technique, and hemodynamics [9,13]. In the Society of Thoracic Surgeons (STS)-reported national rates female gender was indicated as a preoperative risk factor associated with mortality in multivariate logistics regression analysis (*p* = 0.0031) [10]. In a very recent report, [11] higher rates of 30-day mortality, postoperative neurological injury (women 23% vs. men 10.2%; *p* = 0.023), and the long-term risk of death were observed in the women group than in the men group. Women tend to be older and have a higher incidence of stormy preoperative courses resulting in critical presentations or even lethal rupture as well as preoperative higher rate of neurological dysfunction [11]. We counteracted adjustment for baseline differences, which is a critical point in nonrandomized trials, by performing a doubly robust adjustment. In the absence of randomization, this method may reduce the percentage of biased estimates, which may explain why some reports have supported differences in short- or long-term survival rates between the women and men [9,10,11].

The difference in rates of death that we observed in our study was consistent with results that have been previously reported in several studies and registries [5,6,7,12,13,14]. Fukui et al. reviewed 504 patients, 48.6% of which were women, reporting similar operative mortality between the groups (*p* = 0.646). The German Registry for Acute Aortic Dissection Type A (GERAADA) collected data on the surgical procedure of 3380 TAAAD recipients (37% women) enrolled in 56 centers in Germany, Austria, Switzerland, and Luxembourg from July 2006 to June 2015, reporting similar thirty-day mortality between genders (*p* = 0.18). Likewise, evidence from the Nordic Consortium for Acute Type A Aortic Dissection (NORCAAD), a collaborative registry of eight academic cardiothoracic centers in Denmark, Finland, Iceland, and Sweden, reported no difference between the sexes in unadjusted intraoperative death (*p* = 0.17) or 30-day mortality (*p* = 0.99) [12]. Although higher intra-hospital mortality in women was noted in the IRAD registry in the last two reports [9,13], the long-term mortality findings were not confirmed in the last report which did not identify significant differences between the sexes at five-year mortality [13]. Finally, the most recent meta-analysis evaluating sex difference in patients treated surgically for acute type A aortic dissection suggested that women were associated with similar in-hospital/30-day mortality (RR, 1.04; 95% CI, 0.85–1.28; *p* = 0.67) [14]. It should be highlighted that the mean age was homogenous across these studies (61–65 years), as was the percentage of women requiring surgery (31–34%) and the risk factors included in our predictors of in-hospital mortality were reasonable with those disclosed in other registries including co-existing comorbidities (e.g., advanced age) and critical condition at presentation (e.g., mechanical ventilation, preoperative resuscitation) [7,12,13,14].

However, it is important to underline those conflicting results on the average overall operative mortality that emerged in single-center studies with consistent numerical percentage inequality, also in consideration of the extension of the surgical approach, oscillating between values higher than 20% [19] and 24% [20] and values lower than 5.5% [21]. These discrepancies could be reflected in the assessment of operative mortality in gender differences. Recently, Lau et al. [22] observed an average operative mortality of 5.6% lower than that reported in other centers of excellence, although a sex-based difference was not evaluated. Considering the entire population, the authors found no substantial differences between patients who underwent conservative repair with root sparing or hemiarch surgery versus those who received more extensive repair with root replacement and/or complete aortic arch. However, the lack of assessment of operative mortality by type of operation emerges because no comparisons between genders were provided, despite recent IRAD registry reports that women were significantly older and had more often presented later for TAAAD than men (*p* = 0.008) with more frequent presentations with coma/altered mental status, whereas pulse deficits were less common [13]. Notably, this discrepancy emerges among high-volume aortic centers of excellence [21,22,23,24,25] where operative mortality in acute type A aortic dissection with a complex repair is approximately half of that reported in the German and Nordic consortium registries, respectively [2,3]. It has been suggested that high-volume centers with the most experience in aortic surgery improve early outcomes by having the most effective surgical procedure in a specific patient cohort [26,27,28]. Therefore, having a larger volume of data from these centers of excellence on the gender difference in short- and long-term mortality may be of great value in the management of Type A Acute Aortic Dissection in women.

Our results reflect that the location of entry tear was equally distributed among women and men. As previously mentioned, women underwent a lesser proportion of total root replacement, complete or partial arch, and elephant trunk procedures although this was not statistically significant (all *p* > 0.05). We observed that intimal tear resection with prosthetic replacement of the ascending aorta with or without hemiarch implantation is still the most commonly performed operation for type A aortic dissection in women [5,7,9,11,12,13]. As such, our evidence suggested that the extent of surgical repair with the different involvement of ascending aorta or hemiarch or total arch replacement procedure was mostly dictated by surgical evaluation of aortic pathology, and surgical repair was directed at the excision of the dissection entry tear. In particular, an entry tear located in the lesser curvature of the aortic arch was treated with a conservative surgical approach by using an interposition graft with the hemiarch implant. Instead, a more extensive surgical approach with the involvement of the aortic arch, although less frequently in women, was advocated by the presence of the entrance tear located near the supra-aortic branches or was guided by the surgeon’s discernment, based on the patient’s preoperative clinical status and the severity of the involvement of the aortic arch.

In our study, we report 15.1% of women who received total root replacement procedures with no impact on operative mortality. Although aortic root surgery in the context of Type A Acute Aortic Dissection may be constrained by greater technical complexity, other series with a larger number of patients also reported equivalent operative mortality [7,9,11,12,13,29,30]. It has been suggested that total aortic root replacement is recommended in patients who experienced destruction, concurrent root aneurysm, bicuspid aortopathy, or a history of connective tissue disease [31,32]. Importantly, in our series the bicuspid aortic valve was not noted in women. Recently, Lau and colleagues suggested that extensive repair including root replacement and/or total arch was a predictor of later reoperation as compared to the conservative approach that comprised of a root-sparing or hemiarch procedure (*p* = 0.01) [22].

The present analysis revealed that patients who received an extensive procedure involving the total aortic arch replacement procedure were more likely to experience a negative event. Although total arch replacement has been proven to be a safe procedure in the elective setting, there is still an ongoing debate on surgical mortality and morbidity and its long-term benefit in the context of TAAAD [33]. Several studies suggest conflicting data with significant metric variations regarding operative mortality and permanent neurological deficit, which results in contradictory findings emerging from the literature [19,20,21,34]. In particular, a report noted increasing operative mortality correlating to the extent of the procedure involving aortic arch replacements, with mortality rates from 9.8% for the ascending aorta only vs. 21.6% for the hemiarch and 28% for the total arch replacement procedures [19]. Similarly, another study reported an operative mortality of 13.4% for TARP and 9.7% in hemiarch repair [34]. It should be noted that a higher incidence of permanent neurological deficit was observed in the total arch replacement group (22.7% versus 6.3%) [34]. Two independent studies, however, refute these results. Di Eusanio and colleagues [20] found that patients who underwent a conservative approach had a similar incidence of operative mortality and permanent neurologic deficit to those receiving a total arch replacement (24.1% vs. 22.6% and 9.1% vs. 7.5%). Likewise, Zhang and colleagues [21] who compared the conservative approach including hemiarch implantation with a frozen elephant trunk reported no difference in operative mortality (5.4% vs. 5.7%) or permanent neurologic deficit (1.4% vs. 2.3%).

In our analysis, we found that the cerebral perfusion strategy per se was not identified as an independent risk factor for mortality in woman both for antegrade (40.4% vs. 45.1%; *p* = 0.679) or retrograde (17.7% vs. 17.6%; 1000) technique. We observed no difference in postoperative mortality in women patients with a cerebral perfusion undergoing hemiarch or arch replacement surgery (16.3% vs. 13.7%; *p* = 0.833) or without cerebral perfusion receiving aortic root replacement (16.3% vs. 11.7%; *p* = 0.583). Hypothermic circulatory arrest duration in the women cohort was also not identified as a risk factor for operative mortality (median IQR 29.00 vs. 26; *p* = 0.912). Our results are corroborating with other registries (e.g., UK, STS and GERAADA) that have disclosed no association between cerebral perfusion strategies and outcomes [13,29,30]. It is reasonable to assume that a number of factors support this observation. For example, cerebral perfusion could be directed to patients undergoing a more extensive repair with a longer circulatory arrest time such as in the case of an extension of the procedure involving arc replacement. The choice of brain protection strategies is primarily tailored to the experience of the surgeon and his specific skills as well as the characteristics of the patient and the degree of surgical resection. The benefit of cerebral perfusion becomes apparent when the analysis is focused on patients requiring complex procedures, with longer circulatory arrest time (>30 min) [35,36,37,38,39].

Our results reflect the postulation that total arch replacement procedures should be considered while taking into account the patient’s preoperative status and after examining the lesion to be treated. Compared to other experiences [5], our cohort showed a lower percentage of women undergoing arch repair (15.6%). This trend may suggest a degree of conservativism among most surgeons preferring the less complex procedures such as the interposition of a prosthesis with or without hemiarch implantation instead of pushing for more technically demanding repairs when this option is reasonable, although it should be noted that women in our cohort were significantly older than the men. Rylski and colleagues [13] alongside Huckaby and colleagues reported a similar rate of operative strategy regarding the surgical approach involving a total arch replacement procedure in women (13.5% vs. 15.2), while Chemtob and colleagues and Suzuki and colleagues noted that only 4.0% of women received a TARP procedure.

Concerns about the anatomical extent of the lesion in women have suggested that the degree of aortic damage and critical clinical condition at the time of hospital admission, but not the proportion of surgical repair, affect patient outcome. In fact, by univariable analyses and multivariable logistic regression model, we observed that in women preoperative glomerular filtration rate and arterial lactate level were factors associated with a reduction or an increase in operative mortality. Women receive conservative intervention with reduced operative times which are of considerable importance in causing an increase in the risk of malperfusion and worsening of clinical conditions.

This could explain the inherent bias that may direct surgeons’ choice towards a conservative procedure in patients with comorbidities such as elderly women versus choosing a more aggressive surgical option in lower-risk young male patients. Conservative surgery may leave a false circulating lumen which may promote postoperative visceral malperfusion. We have reported that postoperative mesenteric ischemia was a factor associated with in-hospital mortality at univariate and multivariable Cox regression for operative mortality in women. (*p* = 0.003). The GERAADA registry noted other differences between males and females [13]. This included the increased incidence of Marfan’s syndrome, younger age at presentation, and increased incidence of visceral and renal malperfusion in men indicating more extensive disease. Similar outcomes were noted in the NORCAAD study with shorter operative times reflecting the extent of disease and type of repair undertaken [12]. Thus, another criterion to be taken into consideration is that the evaluation of the extent of the disease remains to be established more distinctly and in common agreement. For example, some criteria may consider an assessment of entry point versus size versus extent of dissection, all of which play a role in influencing the best treatment option and outcomes.

Several limitations have been identified in our study. We attempted to match the groups using inverse probability treatment weighting by propensity score; however, this may not completely nullify the differences between the groups. The later age of presentation of women does not take into account their socioeconomic background and frailty which may explain the conservatism among operating surgeons given that previous studies have shown poorer outcomes in this cohort of patients [40,41,42]. Matching may negate some of the differences, especially in the absence of randomization in studies such as this one. This study is also limited by its retrospective nature. Although surgeon’s preferences dictate many of the decision-making processes, this may reflect the multicenter nature of the study and indirect assessment of unknown confounders such as frailty. Patients who were operated on had to initially survive transfer to the tertiary center offering surgery, and this may therefore overlook patients who may have died before admission which have not been captured by the registry.

## 5. Conclusions

This matched analysis highlights the subtle differences between the characteristics of men and women presenting with acute type A aortic dissections and the postoperative outcomes. The advancing age of female patients alongside raised preoperative level of arterial lactate may account for the increasing preponderance among surgeons to perform more conservative surgery compared to their younger male counterparts, although post-operative survival was similar between the groups. Any risk-stratifying methods should include gender to identify high-risk patients to ensure early intervention or referral to tertiary centers with expertise in managing aortic dissections especially if extensive surgery is required. Further studies comparing outcomes of surgery between men and women stratified by age can further elucidate any differences in surgical decision making between the genders.

## Figures and Tables

**Table 1:** Preoperative clinical and operative characteristics of the women and men patients.

Variables	Woman (N = 192)	Man (N = 441)	*p* Value	SMD
Age (median [IQR])	72.00 [61.00, 78.00]	62.00 [54.00, 72.00]	<0.001	0.555
Weight, kg (median [IQR])	65.00 [59.00, 75.00]	81.00 [74.00, 90.00]	<0.001	0.981
Height, cm (median [IQR])	163.00 [160.00, 168.00]	175.00 [170.00, 180.00]	<0.001	1.589
Obesity (%)	25 (13.0)	77 (17.5)	0.201	0.124
eGFR, mL/min/1.73 m^2^ (median [IQR])	61.00 [49.00, 77.00]	77.00 [57.00, 89.00]	<0.001	0.508
Haemoglobin, g/L (median [IQR])	115.00 [104.00, 125.00]	128.00 [110.00, 140.00]	<0.001	0.543
Arterial lactate, mmol/L (median [IQR])	1.30 [0.90, 2.30]	1.40 [1.00, 2.60]	0.262	0.007
Family history of aortic dissection or aneurysm (%)	15 (7.8)	22 (5.0)	0.227	0.116
Prior cardiac surgery (%)	10 (5.2)	11 (2.5)	0.131	0.141
Hypertension (%)	151 (78.6)	347 (78.7)	1.000	0.001
Diabetes (%)	15 (7.8)	24 (5.4)	0.337	0.095
Stroke (%)	4 (2.1)	10 (2.3)	1.000	0.013
Pulmonary disease (%)	15 (7.8)	18 (4.1)	0.081	0.158
Extracardiac arteriopathy (%)	8 (4.2)	14 (3.2)	0.696	0.053
Poor mobility (%)	13 (6.8)	36 (8.2)	0.659	0.053
Recent myocardial infarction (%)	5 (2.6)	14 (3.2)	0.894	0.034
Systolic pulmonary artery pressure (%)			0.477	0.162
<30 mmHg	117 (60.9)	239 (54.2)		
<31 mmHg	1 (0.5)	1 (0.2)		
>55 mmHg	3 (1.6)	5 (1.1)		
30–55 mmHg	21 (10.9)	54 (12.2)		
Bicuspid aortic valve (%)	0 (0.0)	12 (2.7)	0.047	0.237
Cardiogenic shock requiring inotropes (%)	16 (8.3)	52 (11.8)	0.249	0.115
Cardiac tamponade (%)	38 (19.8)	69 (15.6)	0.244	0.109
Preoperative intubation (%)	61 (31.8)	122 (27.7)	0.341	0.090
Any malperfusion excluding myocardial malperfusion (%)	41 (21.4)	109 (24.7)	0.416	0.080
Tear in the aortic root (%)	45 (23.4)	136 (30.8)	0.072	0.167
Tear in the ascending aorta (%)	116 (60.4)	229 (51.9)	0.059	0.172
Tear in the aortic arch (%)	20 (10.4)	70 (15.9)	0.092	0.162
CABG (%)	16 (8.3)	40 (9.1)	0.882	0.026
Aortic root replacement (%)	29 (15.1)	106 (24.0)	0.016	0.227
Total or partial aortic arch repair (%)	30 (15.6)	103 (23.4)	0.037	0.196
Antegrade cerebral perfusion (%)	80 (41.7)	200 (45.4)	0.441	0.074
Retrograde cerebral perfusion (%)	34 (17.7)	83 (18.8)	0.826	0.029
Myocardial ischemic time, min (median [IQR])	91.00 [64.50, 133.50]	108.00 [77.00, 152.00]	0.005	0.179
Cardiopulmonary bypass time, min (median [IQR])	164.00 [119.00, 222.00]	186.00 [128.00, 260.00]	0.012	0.163
Hypothermic circulatory arrest duration, min (median [IQR])	28.00 [15.50, 41.50]	30.00 [5.00, 48.00]	0.529	0.143

Abbreviations; CABG, coronary artery bypass grafting; eGFR, estimated glomerular filtration rate; SMD, standardized mean difference.

**Table 2:** Early outcomes and the doubly robust matching estimators for confounding adjustment of the female and male patients.

Variables	Overall Series			Doubly Robust Adjustment ^§^		
	Female Gender N = 192	Male Gender N = 441	*p* Value	Odds Ratio	95% CI	*p* Value
Stroke (%)	25 (13.0)	57 (12.9)	0.481	0.928	0.521–1.653	0.800
Paraplegia (%)	9 (4.7)	14 (3.2)	0.987	1.379	0.563–3.380	0.483
Tetraplegia or tetraparesis (%)	1 (0.5)	4 (0.9)	0.337	0.492	0.054–4.452	0.529
Laryngeal nerve palsy (%)	3 (1.6)	2 (0.5)	0.569	4.997	0.822–30.393	0.081
Mesenteric ischemia (%)	11 (5.7)	19 (4.3)	0.861	0.936	0.423–2.071	0.871
Sepsis (%)	33 (17.2)	80 (18.1)	0.168	0.866	0.511–1.470	0.595
Dialysis (%)	16 (8.3)	55 (12.5)	0.241	0.504	0.271–0.939	0.031
Atrial fibrillation (%)	47 (24.5)	88 (20.0)	0.583	1.098	0.712–1.693	0.673
Reoperation for intrathoracic bleeding (%)	17 (8.9)	47 (10.7)	1.000	0.683	0.367–1.270	0.229
Deep sternal wound infection/mediastinitis (%)	6 (3.1)	13 (2.9)	0.746	1.062	0.368–3.071	0.911
IABP (%)	4 (2.1)	6 (1.4)	0.971	1.773	0.478–6.572	0.392
VA ECMO (%)	7 (3.6)	18 (4.1)	0.705	0.560	0.224–1.402	0.216
ICU stay, days (median [IQR])	10.00 [4.00, 19.00]	9.00 [3.00, 20.25]	0.483	−0.954 ^§^	1.393 ^§^	0.494 ^§^
In-hospital mortality (%)	51 (26.6)	104 (23.6)	1.000	0.745	0.491–1.130	0.166

Abbreviations; CI, confidence interval; IABP, intra-aortic balloon pump; ICU, intensive care unit; N.A., not applicable; VA ECMO, venoarterial extracorporeal membrane oxygenation. Reference for the events: Female cohort. ^§^ Linear regression has been expressed as standard regression coefficient, standard error and *p* value.

**Table 3:** Univariate and multivariable analyses for operative mortality of women patients.

Variables	Univariate Analysis			Multivariable Analysis			
	No Event (N = 141)	Operative Mortality (N = 51) *	*p* Value	Beta-Coefficient	OR	95% CI	*p* Value
Baseline and operative characteristics							
Age (median [IQR])	69.00 [59.00, 78.00]	76.00 [72.00, 80.00]	<0.001				
Weight, kg (median [IQR])	65.00 [59.75, 75.25]	63.50 [58.00, 72.25]	0.431				
Height, cm (median [IQR])	163.00 [159.75, 169.00]	161.00 [160.00, 167.00]	0.349				
Obesity (%)	20 (14.2)	5 (9.8)	0.580				
eGFR, mL/min/1.73 m^2^ (median [IQR])	65.00 [58.00, 82.00]	50.00 [37.00, 61.00]	<0.001	−0.049	0.952	0.929–0.976	<0.001
Hemoglobin, g/L (median [IQR])	117.50 [106.75, 125.00]	109.00 [100.75, 123.50]	0.112				
Arterial lactate, mmol/L (median [IQR])	1.10 [0.90, 2.00]	2.10 [1.20, 4.50]	<0.001	0.384	1.468	1.133–1.901	0.004
Family history of aortic dissection or aneurysm (%)	12 (8.5)	3 (5.9)	0.768				
Prior cardiac surgery (%)	8 (5.7)	2 (3.9)	0.909				
Hypertension (%)	108 (76.6)	43 (84.3)	0.340				
Diabetes (%)	10 (7.1)	5 (9.8)	0.754				
Stroke (%)	2 (1.4)	2 (3.9)	0.617				
Pulmonary disease (%)	6 (4.3)	9 (17.6)	0.006				
Extracardiac arteriopathy (%)	5 (3.5)	3 (5.9)	0.759				
Poor mobility (%)	9 (6.4)	4 (7.8)	0.976				
Recent myocardial infarction (%)	1 (0.7)	4 (7.8)	0.026				
Systolic pulmonary artery pressure (%)			0.455				
<30 mmHg	81 (57.4)	36 (70.6)					
<31 mmHg	1 (0.7)	0 (0.0)					
>55 mmHg	2 (1.4)	1 (2.0)					
30–55 mmHg	18 (12.8)	3 (5.9)					
Bicuspid aortic valve (%)	0 (0)	0 (0)	NA				
Cardiogenic shock requiring inotropes (%)	7 (5.0)	9 (17.6)	0.012				
Cardiac tamponade (%)	19 (13.5)	19 (37.3)	0.001				
Preoperative intubation (%)	38 (27.0)	23 (45.1)	0.027				
Any malperfusion excluding myocardial malperfusion (%)	21 (14.9)	20 (39.2)	0.001				
Tear in the aortic root (%)	33 (23.4)	12 (23.5)	1.000				
Tear in the ascending aorta (%)	84 (59.6)	32 (62.7)	0.818				
Tear in the aortic arch (%)	14 (9.9)	6 (11.8)	0.920				
CABG (%)	12 (8.5)	4 (7.8)	1.000				
Aortic root replacement (%)	23 (16.3)	6 (11.8)	0.583				
Total or partial aortic arch repair (%)	23 (16.3)	7 (13.7)	0.833				
Antegrade cerebral perfusion (%)	57 (40.4)	23 (45.1)	0.679				
Retrograde cerebral perfusion (%)	25 (17.7)	9 (17.6)	1.000				
Myocardial ischemic time, min (median [IQR])	91.50 [66.75, 126.50]	91.00 [60.00, 158.50]	0.912				
Cardiopulmonary bypass time, min (median [IQR])	156.50 [119.00, 218.25]	182.00 [122.50, 252.50]	0.255				
Hypothermic circulatory arrest duration, min (median [IQR])	29.00 [17.00, 42.00]	26.00 [10.00, 41.25]	0.596				
Postoperative events							
Stroke (%)	13 (9.2)	12 (23.5)	0.018				
Paraplegia (%)	5 (3.5)	4 (7.8)	0.391				
Tetraplegia or tetraparesis (%)	0 (0.0)	1 (2.0)	0.595				
Laryngeal nerve palsy (%)	3 (2.1)	0 (0.0)	0.696				
Mesenteric ischemia (%)	1 (0.7)	10 (19.6)	<0.001	3.489	32.742	3.361–319.017	0.003
Sepsis (%)	23 (16.3)	10 (19.6)	0.750				
Dialysis (%)	8 (5.7)	8 (15.7)	0.055				
Atrial fibrillation (%)	40 (28.4)	7 (13.7)	0.058				
Reoperation for intrathoracic bleeding (%)	14 (9.9)	3 (5.9)	0.559				
Deep sternal wound infection/mediastinitis (%)	3 (2.1)	3 (5.9)	0.395				
IABP (%)	2 (1.4)	2 (3.9)	0.617				
VA ECMO (%)	2 (1.4)	5 (9.8)	0.021				
Constant				0.884			

Abbreviations; CABG, coronary artery bypass grafting; CI, confidence interval; eGFR, estimated glomerular filtration rate; IABP, intra-aortic balloon pump; OR, odds ratio; VA ECMO, venoarterial extracorporeal membrane oxygenation. Reference for the events: Female cohort. * In-hospital mortality: 51/192 (26.6%) patients. NA, not applicable.

## Data Availability

Drs. Nappi, Salsano, and Fiore had full access to all of the data in the study and take responsibility for the integrity of the data and the accuracy of the data analysis. The data underlying this article will be shared on reasonable request to the corresponding author.

## Supplementary Materials

The following supporting information can be downloaded at: https://www.mdpi.com/article/10.3390/jcdd10040147/s1, Figure S1: Covariate balance plot in weighted samples for the female and male patients; Figure S2: Love plot graphically displaying covariate balance before and after adjusting; Figure S3: ROC curve of the propensity scores; Figure S4: Variation of in-hospital mortality over time; Table S1: Covariate balance analyses in weighted samples for the female and male patients.
