# Supplementary material for: Sex-Based Difference in Aortic Dissection Outcomes: A Multicenter Study
Source: J Cardiovasc Dev Dis. 2023 Mar 30;10(4):147. doi: 10.3390/jcdd10040147 (PMC10143202; doi:10.3390/jcdd10040147)

Supplementary Table S1. Covariate balance analyses in weighted samples for the female and male patients.

Variable	Type	Unadjusted sample							Adjusted sample						
		Female 192 pts		Male 441 pts		Balance Measures			Female 145.02 pts		Male 416.54 pts		Balance Measures		
		Mean	Standard deviation	Mean	Standard deviation	Mean difference	Variance ratio	KS	Mean	Standard deviation	Mean	Standard deviation	Mean difference	Variance ratio	KS
Age	Contin.	690.938	123.594	621.497	126.606	-0.5550	10.493	0.2910	641.090	138.369	640.629	126.716	-0.0037	0.8387	0.0750
eGFR	Contin.	624.235	210.973	733.653	219.317	0.5085	10.807	0.0494	696.721	221.654	700.080	226.666	0.0156	10.457	0.0560
eGFR:<NA>	Binary	0.1146	0.3185	0.1247	0.3304	0.0312	.	0.0101	0.1132	0.3168	0.1205	0.3256	0.0226	.	0.0073
Family history of aortic dissection or aneurysm	Binary	0.0781	0.2684	0.0499	0.2177	-0.1156	.	0.0282	0.0654	0.2472	0.0580	0.2338	-0.0300	.	0.0073
Prior cardiac surgery	Binary	0.0521	0.2222	0.0249	0.1560	-0.1414	.	0.0271	0.0358	0.1857	0.0348	0.1833	-0.0051	.	0.0010
Hypertension	Binary	0.7865	0.4098	0.7868	0.4095	0.0010	.	0.0004	0.7999	0.4001	0.7899	0.4074	-0.0244	.	0.0100
Diabetes	Binary	0.0781	0.2684	0.0544	0.2268	-0.0954	.	0.0237	0.0471	0.2118	0.0598	0.2371	0.0512	.	0.0127
Obesity	Binary	0.1302	0.3365	0.1746	0.3796	0.1238	.	0.0444	0.1575	0.3643	0.1642	0.3704	0.0185	.	0.0067
Prior stroke	Binary	0.0208	0.1428	0.0227	0.1489	0.0126	.	0.0018	0.0273	0.1630	0.0239	0.1526	-0.0238	.	0.0035
Pulmonary disease	Binary	0.0781	0.2684	0.0408	0.1979	-0.1582	.	0.0373	0.0499	0.2178	0.0468	0.2111	-0.0135	.	0.0032
Extracardiac arteriopathy	Binary	0.0417	0.1998	0.0317	0.1753	-0.0528	.	0.0099	0.0315	0.1746	0.0315	0.1748	0.0004	.	0.0001
Poor mobility	Binary	0.0677	0.2512	0.0816	0.2738	0.0530	.	0.0139	0.0764	0.2656	0.0791	0.2698	0.0102	.	0.0027
Recent myocardial infarction	Binary	0.0260	0.1593	0.0317	0.1753	0.0341	.	0.0057	0.0178	0.1321	0.0305	0.1721	0.0763	.	0.0128

Cardiogenic shock requiring inotropes	Binary	0.0833	0.2764	0.1179	0.3225	0.1151	.	0.0346	0.0880	0.2833	0.1099	0.3128	0.0728	.	0.0219
Intubated at arrival	Binary	0.3177	0.4656	0.2766	0.4473	-0.0899	.	0.0411	0.2869	0.4523	0.2907	0.4541	0.0083	.	0.0038
Any malperfusion excluding myocardial malperfusion	Binary	0.2135	0.4098	0.2472	0.4314	0.0799	.	0.0336	0.2280	0.4196	0.2388	0.4264	0.0256	.	0.0108
Total or partial aortic arch repair	Binary	0.1562	0.3631	0.2336	0.4231	0.1961	.	0.0773	0.1934	0.3949	0.2094	0.4069	0.0406	.	0.0160
Bicuspid aortic valve	Binary	0.0000	0.0000	0.0272	0.1627	0.2365	.	0.0272	0.0000	0.0000	0.0188	0.1357	0.1632	.	0.0188

eGFR, estimated glomerular filtration rate

Supplementary Figure S1. Covariate balance plot in weighted samples for the female and male patients.

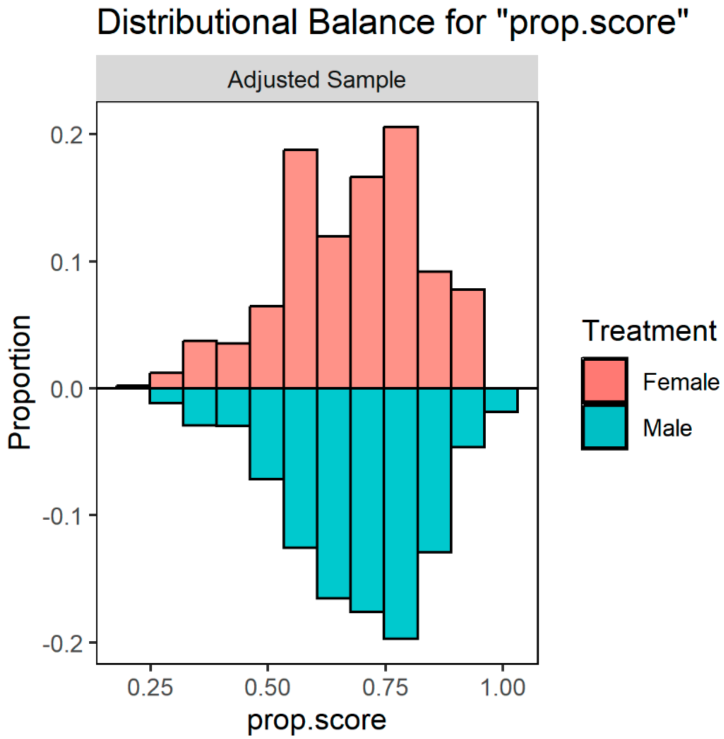

Supplementary Figure S2. Love plot graphically displaying covariate balance before and after adjusting.

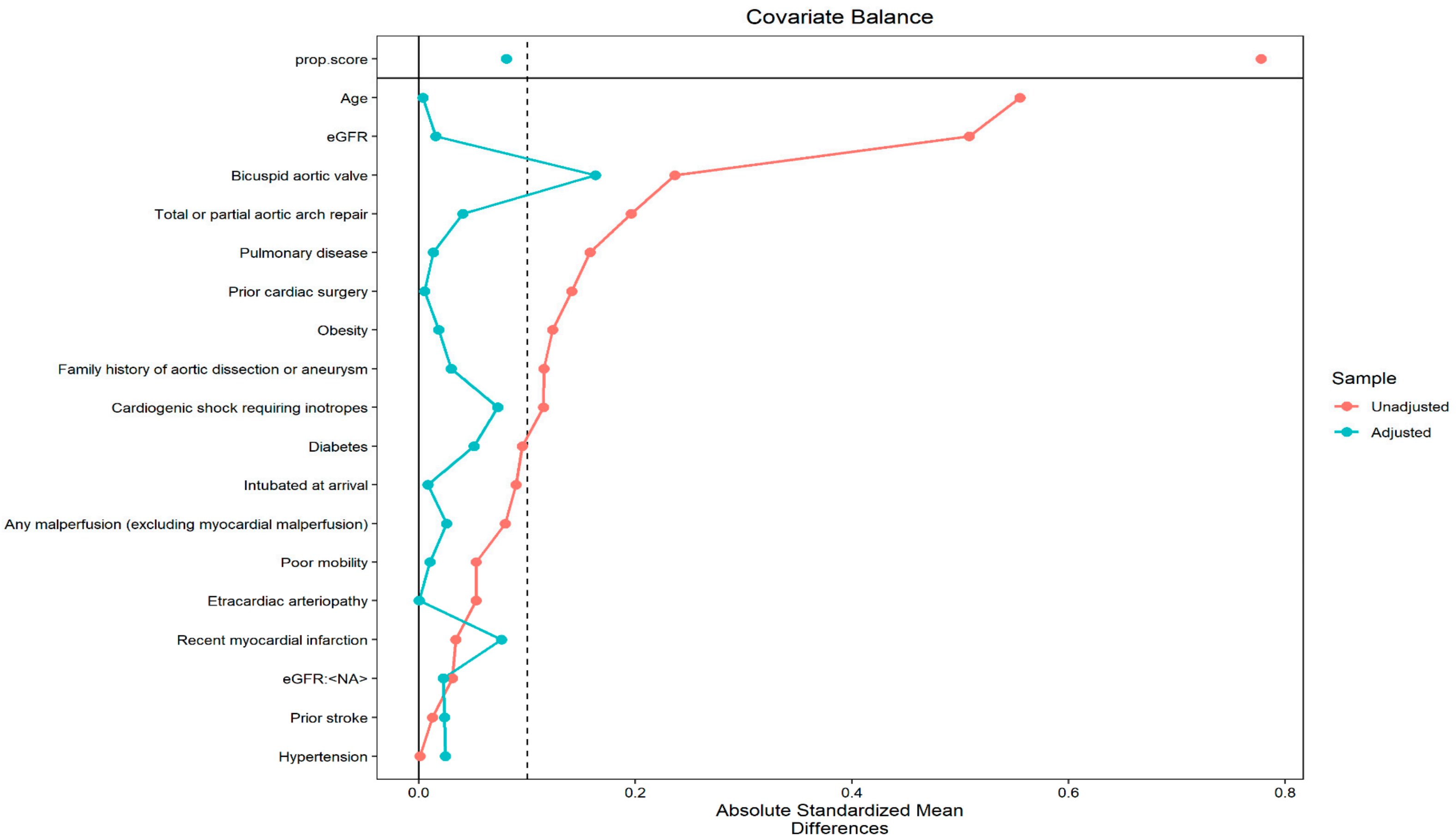

Supplementary Figure S3. ROC curve of the propensity scores.

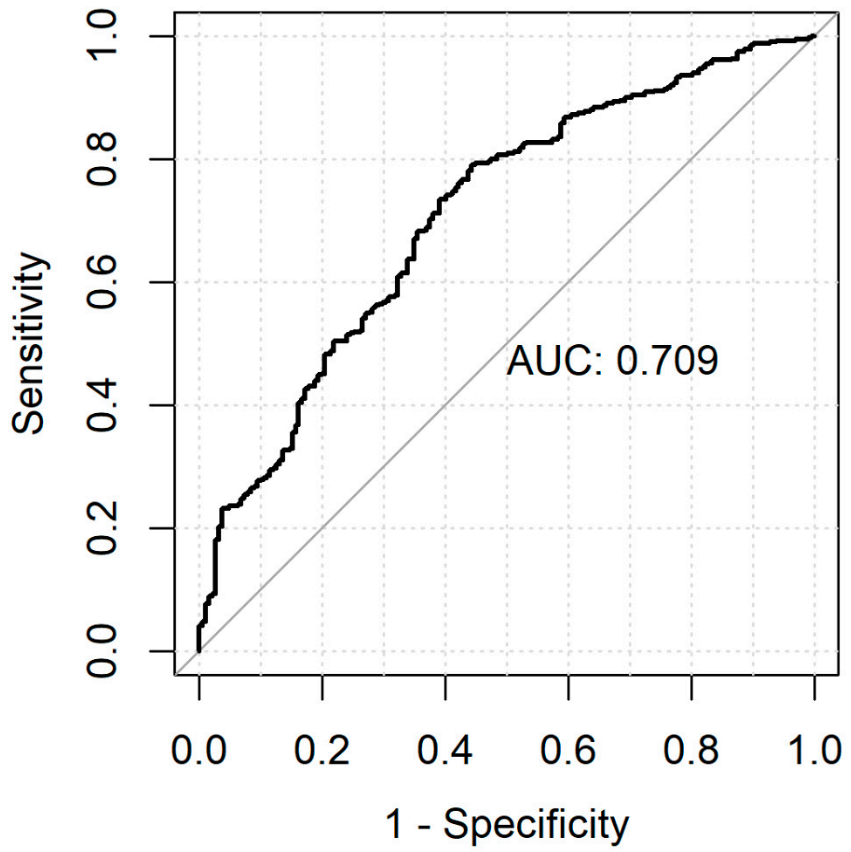

Supplementary Figure S4. Variation of in-hospital mortality over time.

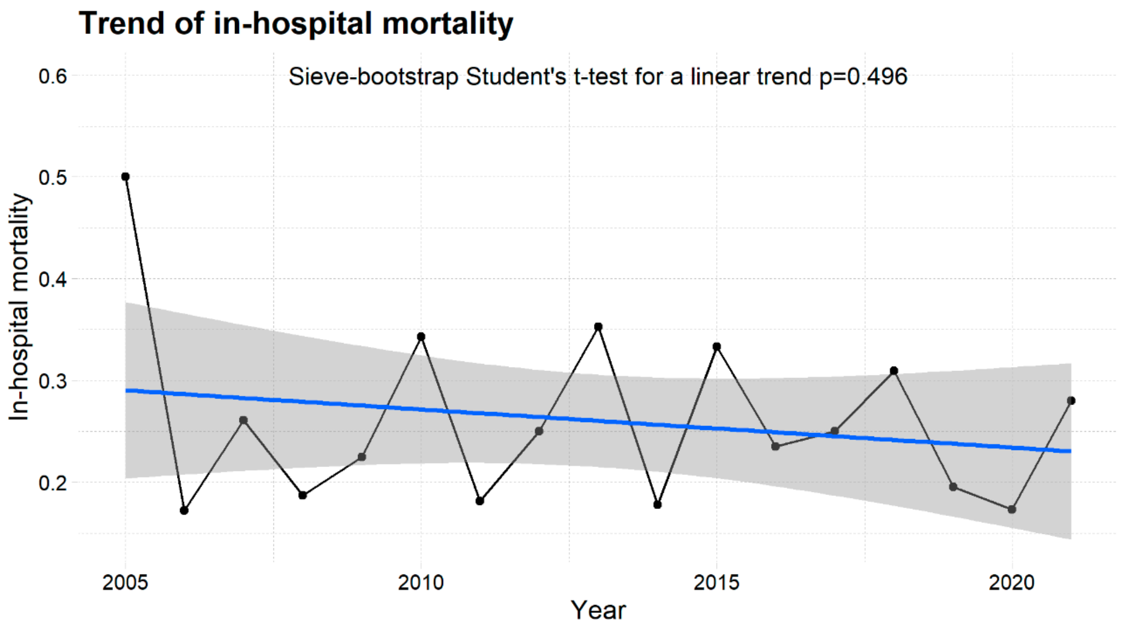

Supplement: Supplementary file 1 [file jcdd-10-00147-s001.zip › jcdd-2296559-supplementary.pdf]
